# The Impact of the Surface Modification on Tin-Doped Indium Oxide Nanocomposite Properties

**DOI:** 10.3390/nano12010155

**Published:** 2022-01-03

**Authors:** Arash Fattahi, Peyman Koohsari, Muhammad Shadman Lakmehsari, Khashayar Ghandi

**Affiliations:** 1 Department of Chemistry, University of Guelph, Guelph, ON N1G 2W1, Canada; afattahi@uoguelph.ca; 2 Department of Chemistry, Faculty of Science, University of Zanjan, Zanjan P.O. Box 45195-313, Iran; koohsaripeiman@gmail.com (P.K.); shadman@znu.ac.ir (M.S.L.)

**Keywords:** surface treatment, density functional theory, molecular dynamics, ITO, nanocomposite

## Abstract

This review provides an analysis of the theoretical methods to study the effects of surface modification on structural properties of nanostructured indium tin oxide (ITO), mainly by organic compounds. The computational data are compared with experimental data such as X-ray diffraction (XRD), atomic force microscopy (AFM) and energy-dispersive X-ray spectroscopy (EDS) data with the focus on optoelectronic and electrocatalytic properties of the surface to investigate potential relations of these properties and applications of ITO in fields such as biosensing and electronic device fabrication. Our analysis shows that the change in optoelectronic properties of the surface is mainly due to functionalizing the surface with organic molecules and that the electrocatalytic properties vary as a function of size.

## 1. Introduction

The applications of metal oxides in catalysis, sensing, electronics, and energy storage have been rapidly developed [1,2,3,4,5]. Among metal oxides, indium tin oxide (ITO) is one of the most important transparent conductive oxides (TCO), due to its unique optoelectronic properties [6,7,8,9,10]. It is an n-type degenerate semiconductor with a band gap in the range of 3.5–4.3 eV [11]. Therefore, it is wildly used as a transparent electrode in optoelectronic devices [9,10,11,12,13]. Emerging new fabrication techniques for synthesizing metal oxides and nanocomposites such as ITO, ZnO/CuO and Al_2_O_3_/ZrO_2_ results in improving the electrical, optical, and catalytic properties of these materials [14,15,16]. As such, the ITO is widely used in different applications such as solar cells, photovoltaic devices, organic light-emitting diodes and biosensors [15,17]. However, there are unanswered questions in the field for surface modification of the ITO and its impact on the ITO physical properties. The motivation of this review is to address these unanswered questions.

Numerous computational and experimental methods have been implemented to investigate surface defects, surface interactions, binding between organic molecules and the surface, and structural properties of nanomaterials such as gold nanoparticles [18], CoBr_2_ nanolayers [19], CdO and ZnO [20] metal oxides and SnO_2_/CdO, ITO and Zn_2_SiO_4_/ZnO nanocomposites [21,22,23,24]. Due to experimental limitations, it is difficult to analyze the interaction of organic molecules and surfaces at the molecular level. For instance, adsorption properties and diffusion mechanisms of molecules on the surface, molecular orientation and conformation, and information regarding specific molecules interacting with surfaces are unclear. As such, several simulation approaches have been extensively used to study the interface of organic molecules and metal oxides [25,26,27].

The first principle calculations have been applied to provide molecular-level insights about the interface of metal oxides and molecules [28] and to study the chemical reactions on the surface for close to three decades [29]. Recently, with new developments in computing power, software and computational algorithms, using the density functional theory (DFT), made these simulations applicable for the study of interface properties [30,31,32]. In recent years, the DFT has also been used to investigate the physical properties of alloys, especially the thermal conductivity of different alloys [33,34,35,36,37].

Modification of conducting metal oxides and the concept of doping are of wide interest [38,39,40,41,42,43]. There are several reports on investigating the interface of biomolecules and metal oxides [29,30,31,32,33,34,35], in particular indium oxide [44,45,46] and titania [47,48]. Recently, studies have been performed on surface treatment of ITO using biomolecules for investigating its electrochemical properties and electronic properties for biosensing, including even faster biosensors, to help fight the current pandemic [6,17,49,50]. However, to completely understand the mechanisms at play for such applications, a detailed, comprehensive computational study of the interface is required. To that end, we focus on reviewing the surface properties of the ITO in the absence and presence of biomolecules by addressing the following questions, analyzing the dataset available from a combination of computational and experimental literature:

— What would be the best methods to study the surface properties of the ITO?
— What are the effects of surface changes on the structural properties at the interface?
— How does the nanostructure affect the electrocatalytic properties of the ITO?

We introduced the most often applied methods used for first-principal investigations to address these questions. After that, the latest computational works performed on the ITO have been compared with the experimental results. The aim is to identify the current gaps in both computational and experimental work on ITO. In addition, new insights based on comparing the experimental and computational works are presented. At the end of this review, we mentioned some of the challenges and opportunities of ITO simulations.

## 2. Computational Methods to Study the Surface Properties of the ITO

To determine the best simulation approach for investigating the surface properties of the ITO, our focus is on the comparison of the theoretical and experimental data. The main structural properties of the ITO that has been studied theoretically so far are optoelectronic properties such as band gap, work function, and charge injection rate [21,22,23,51,52,53,54]. Moreover, the time and size of the computational works are two main factors affecting the cost of the simulation. Therefore, efforts should be made to optimize the computational cost. We first introduce different simulation approaches used to characterize the ITO. Then we will compare the electronic structure of the ITO studied with different computational methods. In the end, the best method can be introduced, considering the cost of the calculations as well as the consistency of the ITO properties with experimental analysis.

### 2.1. Overview of Different Simulation Approaches

The time, cost, and size of the computational studies depend on the number of atoms in the calculation. Generally, to study the interaction of even a small biomolecule such as a small peptide on the surface requires the order of thousands of atoms [17]. Therefore, obtaining a quantum-level analysis of the surface is very difficult. One way to speed up the calculation is by applying a physical model described by an empirical force field [55,56]. This enables predicting the interactions at the atomistic level for large systems using molecular dynamics (MD) simulations [57,58,59]. Another factor for enhancing the simulation is to represent the behavior of the system by sampling all the conformations possible for the molecule being studied. In addition, the model needs to satisfy the interface between all the compounds of the system. This is especially important in the case of studying the interactions of a biomolecule such as peptides on a surface in the presence of water or any other aqueous solution as media [60]. Herein, a model to represent the organic and inorganic materials, as well as the media on the interface between the two materials, is necessary. Therefore, a compromise is usually made to simplify the model. Developing structural models, generating force fields, and providing conformational modeling are challenges in this field. According to a review published by Walsh in 2017, these challenges have been addressed by mainly implying noble face-centered cubic metal structures and, to a lesser extent, metal oxides [61].

Different parameters affect the surface properties of the ITO. In this review article, we focus on the effects of functionalization and nanoscale morphology on the surface of ITO. The relation between these parameters and the surface properties can be investigated using MD simulation and the more expensive electronic structure calculations.

Few works have been performed using MD simulation to study both the interface properties and doping mechanism in ITO [62,63]. For this purpose, MD is first performed to relax the surface structure of the ITO. Relaxing the surface makes atoms to be localized in a local energy minimum in the united cell. After that, properties such as total energy, enthalpy, heat capacity, thermal conductivity are calculated.

The surface modification of the ITO can also be investigated using first-principle calculations based on DFT [52,54,64,65,66]. Structural, optical, and electronic properties such as binding energy, band gap, work function, electronic coupling are the main properties that are calculated using DFT. Properties of ITO in both bulk- and thin-film forms have been studied, and the results are compared with experimental data [23,67,68,69].

#### 2.1.1. MD Simulation

MD is usually used to simulate the shape of large molecules such as proteins [70,71]. It can define the atomic spatial position. It also generates the thermodynamics properties of the molecules. In this approach, solving the classical Newton’s law of motion for the molecule provides the energy of the molecule. For a given molecule, a set of possible atom locations gives a conformational profile. The five most common software for MD simulation are: LAMMPS [72], GROMACS [73], NAMD [74], CHARMM [75], and AMBER [76]. Among these, LAMMPS and GROMACS are open-source software and are widely used by scholars. The main parameters in all the software are Coulomb and Van der Waals interaction forces for atoms, effective charge for each atom, integration time step, and total simulation time. The integration time step is mostly 1 fs in calculations. The MD simulation takes place mostly under two conditions: constant pressure and temperature (NPT) and constant volume and temperature (NVT). The well-known thermostat and barostat are available for using them in NPT or NVT ensembles such as Nosé–Hoover and Berendsen in each ensemble from pico- to nano-seconds [77].

To calculate the inter and intramolecular forces between all atoms of molecules in any molecular simulation, it is necessary to utilize the proper force fields. A force field is a collection of mathematical equations describing the dependence of the energy of a system on the coordinates of its particles. The constants in the mathematical equations are typically obtained either from ab initio or semi-empirical quantum calculations or by fitting a mathematical model to experimental data. There are many force fields available in the literature. Potentials that are usually used for interaction forces are: Buckingham, Lennard-Jones, and Born–Mayer–Huggins. Typical expression for a force field contains two parts: intramolecular terms (repulsive and Van der Waals interactions) and intermolecular terms (bond stretching, angle bending, and dihedral and improper torsions). Currently, there are a lot of force fields available for calculations. The most popular ones are: CHARMM, AMBER, GROMOS, OPLS, COMPASS, DREIDING, etc. [78]. It is important to parametrize the force field for complex works. Moreover, to develop a new force field, it is required to model the system energy and select a function. A data set has to be selected in order to determine all the parameters of the function. Finally, optimizing the parameters and validating the final set of parameters will take place [56].

Utsuno et al. studied the tin doping mechanism in indium oxide structure and its effects on ITO conductivity using MD. First, Sn is substituted for In, then oxygen atoms and Sn atoms are relaxed. Moreover, additional oxygen is required to provide a charge balance. As such, Sn potential energy derived by MD provides information regarding the behavior of substituted Sn and interstitial oxygen [79]. Lorenzoni et al. also used MD to simulate the nanoscale morphologies of ITO and organic conductive molecules (Iridium complex Tris DPBIC (3-phenyl-1H-benzimidazol-1-yl-2(3H)-ylidene)-1,2-phenylene)). Their goal was to study the charge injection mechanism at the interface of the metal/organic complex [63]. They performed a two-step, top-down simulation approach. The first step was MD simulations of a nanoscale molecular aggregation phenomenon at the organic/electrode interface. MD simulations consisted of a combination of equilibrium and non-equilibrium approaches. The equilibrium approach was achieved by a run of 100 ps at 300 K for each of the DPBIC molecules when the molecules are in contact with the surface of the ITO. In the non-equilibrium approach, a total of 1 ns run was performed on the system. Here, the organic molecules were equilibrated on the surface of the ITO at different temperatures at different times. They compared kinetic and thermodynamically-controlled growth conditions. For this purpose, they studied the distance between the center of the DPBIC molecule and the surface of the ITO and the orientation angle of the molecule with respect to the slab of the ITO under each condition. Results of angular orientation suggest that preferred orientations are obtained at 0° and 180°.

#### 2.1.2. First-Principal Quantum Calculations

The first principle studies are based on quantum mechanics by solving the Schrödinger equation of many body systems [53]. They use the interaction among nucleus and electrons to study the physical and chemical properties [80,81,82]. Some calculations are based on the Hartree–Fock self-consistent field approach and solving the Schrödinger equation without implying empirical parameters [83]. On the other hand, density functional calculation contains some empirical parameters and fittings. They are based on the number density of particles and electron density that are obtained by finding the ground state energy during solving the Schrödinger equation [84]. To investigate the surface properties of the ITO, the calculated electron density distribution on the surface of materials (surface states) is used to compare with experimental surface characterizations [23,65,66,69,85,86]. In DFT methods, the only electron density is considered among all the aspects of electron-based properties [82]. As such, a lower computational cost is possible. DFT is performed to characterize catalytic and, especially, electronic properties of the ITO such as charge transfer rate and electronic work function modification [23,87,88,89].

#### 2.1.3. Quantum Computational Packages

First-principle studies on ITO has been conducted mostly by using the Vienna ab initio simulation package (VASP) [89,90,91,92]. Other chemical software such as quantum espresso (QE) [93], Spanish initiative for electronic simulations with thousands of atoms (SIESTA) [94], and DMol3 [95] are also used. Harrel et al. [96] used QE to simulate the (001) plane of ITO in rhombohedral structure to study the effects of hydroxylation, calculating the electronic structure performed using periodic boundary conditions, k-point simulating of the first Brillouin zone and plane wave basis set. DFT calculation and geometry optimization of the ITO structure with QE package were also used by Lorenzoni et al. [63]. They studied the electronic coupling and injection rates between ITO and an organic biomolecule. Oxidation of the ITO surface was also conducted by Zhou et al. [21]. The model was based on choosing a crystalline surface with (110) orientation. Electronic structure simulation was performed using the SIESTA package. In contrast to VASP and QE that is based on plane waves, SIESTA is based on a linear combination of atomic orbitals (LCAO) method. In their work, the VASP package was also used to compare the accuracy for some of the parameters. Calculating cohesive energy indicated no significant difference between using two packages [21]. Another significant paper that calculated the electronic and optical properties of bulk ITO has used Dmol3 package [65]. By changing the dopant level, Brewer et al., investigated the changes in optoelectronic properties.

As far as our research shows, QE is the most used package after VASP for the DFT calculation of the ITO. It seems that the calculations are performed at a faster rate in VASP. A comparison can be based on calculating silicone supercell with 31 atoms using VASP 5.3.3 and QE 5.0.1. Running with 16 cores and 8 cores, VASP is 6% faster than QE in both cases [97]. VASP comes with a pseudopotential library and is proprietary software. On the other hand, QE has very good support. It is license-free and accessible to everyone. One of the main disadvantages of QE is the need for large computational resources (too much RAM) for calculations. For calculations of vibrational properties, QE contains implementations of density functional perturbations theory (DFPT), whereas VASP uses another third-party software. There are many third-party packages for post-processing that support VASP [98]. Among those, atomic simulation environment (ASE) is the most popular one. ASE is software written in the python language. The program is used to analyze atomistic simulations. In ASE, the energy, force, stress and other physical properties are calculated using a force field with the same interface. The role of ASE is to provide modules for the computational works, including, but not limited to, optimization and MD calculations [99]. VASP supports more functionals than other software. VASP has its own pseudopotentials (PPs) that include some ultrasoft pseudopotential support. It also uses norm-conserving and Bloechl PAW pseudopotentials. Moreover, ill-behaved PP are easily spotted and can be replaced by improved potentials. There are well-tested PPs for VASP, whereas proper PP for others must be chosen as there are many for the same element [98,100,101]. SIESTA is another license-free package that uses localized basis sets. Similar to SIESTA, DMol3 is based on localized basis sets either. In packages with localized basis sets, additional approximations are added. However, studies have shown that the parameters calculated using both techniques are pretty much similar. Moreover, performing simulations using basis sets is faster and cheaper than using plane waves [102].

### 2.2. Electronic Structures

#### 2.2.1. Modeling the Surface

To construct the ITO structure in the form of bulk and thin film, typically indium oxide (32 indium and 48 oxygen atoms) unit cell containing 80 atoms (16 In_2_O_3_) is selected [103]. This is consistent with experimental results performed for identifying the indium oxide crystal structure [104]. Based on the percentage of tin atoms derived from experiments which is 4.3% to 14%, between 1 to 12 atoms of In are substituted by Sn atoms [105]. Generally, to maintain the electroneutrality of the ITO, extra oxygen atoms will be incorporated into the system [87].

Determining crystal structure and crystalline surface orientation is based on the experimental work to find the most abundant slab. The X-ray diffraction (XRD) analysis of ITO reveals that all the films are polycrystalline with a body-centered cubic (BCC) structure. For a higher tin doping level, rhombohedral (rh) structure emerges as well. The highest intensity is attributed to (222) and (001) planes for BCC and rh, respectively [106,107,108]. These planes are predominant among all the reflection planes for the orientation of the ITO. However, to simulate the slab model of the ITO with BCC crystal structure, (111) and (100) are chosen as the most thermodynamically stable surfaces in most of the studies [64,66,88,91,96,109,110]. As such, computational works have to be performed to investigate the differences between cleaving the cubic crystal along different surfaces.

The surface model in the ITO unite cell is terminated either with In/Sn or O atom. The first case is metal terminated, and the second one is called oxygen terminated. Based on the experimental work, the ITO surface is oxygen terminated. The reason is that the topmost ITO surface is often treated with ozone and oxygen plasma in the fabrication process of the ITO. Moreover, the surface model typically contains 3 to 6 alternating layers. The number of layers to be relaxed in the case of surface modification does not affect the adsorption of molecules on the surface. This is due to the very small difference in the calculated adsorption energies after the relaxation of different layers [21]. For example, it is shown that the difference in the calculated adsorption energies between relaxations of the top two and four layers is less than 0.06 eV [103]. The interlayer space between slabs is chosen to be more than 10 Å [64,68,87,103,106]. To provide this space, the distance between slabs needs to be more than the lattice constant of the ITO. Considering the BCC structure, the experimental value for the lattice parameter of the ITO with 10 wt% tin is 10.1288 Å [108,111,112,113]. Therefore, any distance less than the measured values for the lattice parameter will not provide a realistic interlayer space between slabs. The Sn doped atoms are randomly placed between the slabs. The above-mentioned vacuum space provides a thickness of less than 5 nm for the ITO structure. Due to the exponential increase in the computational time by increasing the number of atoms, a limit of 3 nm for the thickness is used. Finally, to have a proper convergence for the structure, a cut-off energy between 300 to 500 eV is considered [23,30,52,62,69,86,87,114]. Using the above parameters, the geometry and crystallographic parameters would be in good agreement with the experimental data.

#### 2.2.2. Calculating Surface Energy

There are several approaches to estimate the energy of a material based on its electron distribution by splitting the energy into several functionals [115]. One approach is the local density approximation (LDA), which approximates the exchange-correlation energy in DFT. Two main aspects of LDA are: (1) exchange-correlation energy per particle only depends on the local density or electronic density at each point in space, and (2) LDA is derived from core-electron interaction of homogeneous electron gas (HEG) systems. HEG is a model where electrons interact in a solid system where positive charges of the solid are uniformly distributed in the space. As such, LDA assumes that the contribution of each point on the total exchange-correlation energy only depends on its own density. The total exchange-correlation energy is calculated by the sum of energy for each point. Overall, LDA underestimates the band gaps and bond lengths of solids and overestimate the binding energies [102]. Another way is to include the gradient of the exchange into the system [116]. E.g., the exchange-correlation energy can be described by Perdew–Burke–Ernzerhof (PBE) functional, which belongs to the class of generalized gradient approximation (GGA) functionals [70,117]. In terms of exchange-correlation, GGA is more accurate than LDA. PBE is popular due to providing reliable results. It provides reasonable bond lengths and angles within 0.02 Å and 2 degrees, respectively [117]. On the other hand, unlike exchange hybrid correlation functionals, PBE (and any GGA) is known to underestimate the highest occupied molecular orbital and the lowest unoccupied molecular orbitals (HOMO–LUMO) gaps. Therefore, hybrid approximations such as B3LYP are better suited for calculating electrical and optical properties [118,119]. B3LYP includes exchange as well as GGA corrections. Hybrid functionals are generated by incorporating the Hartree–Fock (HF) exchange in the GGA functional. The disadvantage of hybrid functionals such as B3LYP is their high cost compared to GGA’s that can use a plane wave basis set such as those in VASP. Therefore, currently, they cannot reasonably be used for the calculation of having heavy atoms such as Sn [120,121,122]. The projected augmented wave (PAW) is used to combine the PBE and plane waves. In PAW, all electrons scattering properties are reproduced over a wide range of energies [123,124]. The obtained results are in good agreement with experimental results on the ITO in all above-mentioned DFT studies.

## 3. Effects of Surface Modification on the Structural Properties of ITO

Various experimental and theoretical studies have probed surface modification of the ITO with organic and inorganic molecules. The focus of researchers has been on enhancing the optoelectronic properties of the ITO. The work function, density of states and band gap of the ITO are the main parameters investigated by scholars. In this section, we describe the interactions between molecules and ITO surface atoms. By analyzing the effects of different parameters on optoelectronic properties, the mechanisms that affect these properties will be explained. Moreover, the effects of different molecules on the properties of the ITO are compared with each other. This will result in optimizing the modification mechanism and determining the compound that has the highest impact on the ITO properties.

### 3.1. Effects of Surface Modification on Work Function

Indium tin oxide as a transparent conductive oxide (TCO) is widely used in optoelectronic devices such as light-emitting diodes (LED) because of its high electrical conductivity and good transparency in invisible light [125,126,127]. Modification of these materials is of interest to tune the electrical properties such as surface work function. ITO has a work function in the range of 3.2–4.9 eV [128,129]. Increasing the effective surface work function leads to improved charge injection to, or collection from, an organic layer [46,63,88,109]. One method to tune the work function of electrodes is the modification of the surface using organic materials such as carboxylic acids, phosphonic acids (PAs), tetrafluorotetracyanoquinodimethane (F_4_TCNQ), and silanes. Modifying the surface affects the work function by increasing the oxygen vacancies on the surface of the ITO. On the other hand, the hole-injection mechanism is enhanced in the interface due to increasing oxygen vacancies. In addition, the reduction of the barrier between the Fermi level in the ITO and HOMO level of the organic biomolecule increases the conductivity. There are various binding mechanisms for the adsorption of organic molecules on the surface of different transition metal oxides [26]. Different mechanisms are based on the degree of hydrogen bonding and the amount of oxygen bound to the surface. In each mechanism, the orientation of the modifier and subsequently the net surface dipole at the interface changes. As such, the surface work function is changed [54,91,109,130]. For ITO thin films, several parameters in surface morphology such as dipole moment, active surface sites, geometry, and grain boundaries affect the electrical and optical properties of the film. Table 1 provides different parameters and their effects on the work function of ITO studied so far.

#### 3.1.1. Parameters Affecting Work Function

##### 3.1.1.1. Nature of Organic Molecules’ Dipole Moments

**Figure 1:**
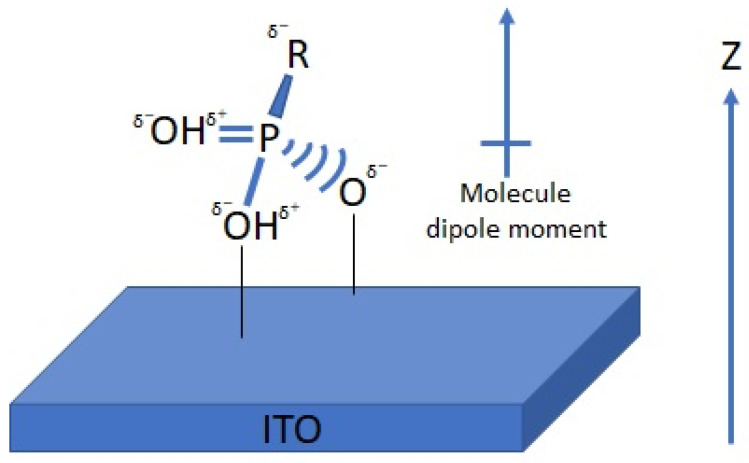
Adsorption of PA on the surface of indium tin oxide. Net dipole moment is aligned with the positive z direction.

To study the relationship between the work function and the surface modification, DFT calculations have been used in several studies [8,23,46,47,72,73,108,109,110,111]. Depositing a thin film of organic or inorganic material on a conductive metal oxide substrate has been shown to affect the effective surface work function. For instance, by functionalizing the surface of the ITO with phosphonic acid, when the direction of the dipole moment is defined to be positive along the positive z-axis (as shown in Figure 1), all molecules present a negative dipole moment (Figure 1). In such an orientation, the calculated potential changes across the organic monolayer are negative [22,46]. Hotchkiss et al. have designed and synthesized a series of fluorinated benzyl PAs [46] to tune the work function by adjusting molecular dipole in different configurations relative to the ITO surface. They selected VASP to perform DFT calculations with the PBE exchange-correlation functional and PAW method. The plane wave basis set with the energy cut-off of 300 eV was used in their simulations. DFT study of the ITO interface and chemisorbed 3,4,5-trifluorophenyl PA molecules has also been performed by Timpel et al. [54]. The calculation was performed using VASP, GGA exchange-correlation PBE functional, PAW method, the plane wave basis set with the 300 eV energy cut-off, and 10^−6^ eV total energy convergence.

These results indicate that attaching a dipolar organic molecule with a negative pole directed away from the surface increases the electrostatic potential energy in the interlayer region and increases the local work function. The opposite is true if the negative pole points toward the surface. On the other hand, experimental works have been performed to investigate the effects of surface treatment using O_2_, N_2_, oxygen plasma, or ozone on the ITO work function. Ultraviolet photoelectron spectroscopy (UPS) and X-ray photoelectron spectroscopy (XPS) indicate the change in the work function and the change in the chemical composition of the ITO surface. Removing carbon contamination by cleaning the surface using oxygen plasma increases the work function significantly according to UPS measurements. Based on XPS results, using the plasma induces a dipole layer on the surface of the ITO, which leads to increasing the work function of the ITO [132,133,134,135,136,137,138,139,140].

##### 3.1.1.2. Coverage Density and Adsorption Site

**Figure 2:**
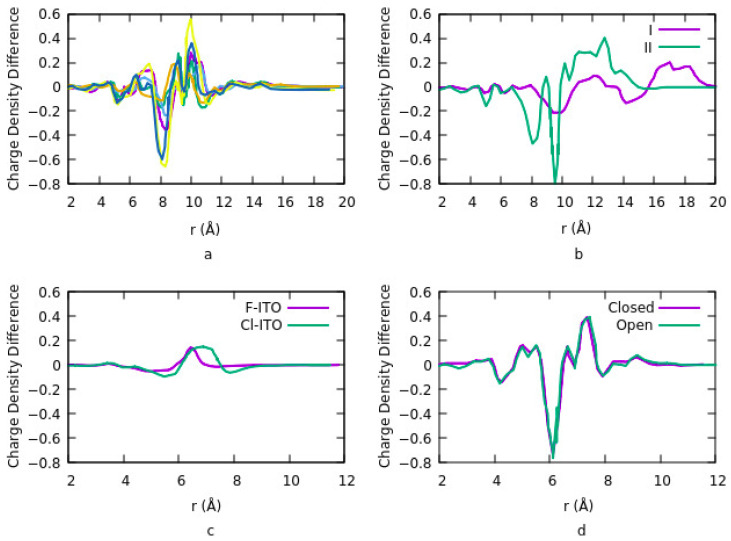
(**a**) Green: site 1, density coverage *n* = 1, Purple: site 4, *n* = 1, Light blue: site 2, 4, *n* = 2, Orange: site 1, 2, *n* = 2, Yellow: site 2, 3, 4, *n* = 3, Dark Blue: site 1, 2, 3, *n* = 3. For *n* = 1, we can see an increase in the electron density near the P atoms in the PA molecules and a decrease near the surface of In atoms bonded to PA. For *n* = 3 the magnitude of a dipole is increased, and different configurations for the higher coverage densities have less impact on the charge density. (**b**) Two different modes of modification of ITO with PA-F_4_TCNQ. I: F4TCNQ is adsorbed on top of carbazole-PA and indirectly binds to ITO, and II: direct binding of F_4_TCNQ to ITO. A sharp decrease in electronic charge density in configuration II indicates the charge transfer from ITO to F_4_TCNQ is higher than ITO to PA. (**c**) Charge density difference for Cl-ITO and F-ITO systems. r = 0 Å refers to the bottom of the ITO slab and for the highest hydrogen atom in the top layer of the ITO r = 9 Å. (**d**) Charge density difference for Open and Closed systems.

DFT calculations have been used on self-assembled monolayers of phosphonic acid chemically bound to the surface of the ITO [110]. VASP method with a GGA exchange-correlation functional of PBE and PAW were used for calculations. Plane wave basis sets with 300 eV cut-off and total convergence of 10^–6^ eV for self-consistent iterations were used. The effects of coverage density or number of molecules per surface and adsorption geometries of phosphonate moiety ($PO_{3}^{2-}$) on the surface, the work-function changes of the interface and the change in density of states have been studied. The surface area of the unit cell was designed in such a way that four different adsorption sites were provided in the unit cell. In this case, the number of PA molecules per surface varied between one to four. This coverage density provides a range from 2.8 × 10^13^ to 1.1 × 10^14^ molecules cm^−2^. The optimum sites were selected for a configuration to have the most uniform distribution at the highest coverage and to provide direct access for the phosphonate moieties to the In/Sn surface atoms. The change in work function is related to charge transfer at the PA-ITO interface. Figure 2a shows the results related to the charge density difference for different geometries and coverages. PA coverage density and binding geometry strongly influence the work function of the ITO surface. For coverage density higher than three molecules per surface, PA adsorption geometry on the ITO is independent of PA charge density. However, the average adsorption energy per molecule is nearly independent of surface coverage for any number of molecules per surface. Thus, intermolecular interactions among organic molecules do not affect the binding strength between the PA molecules and ITO, and the work function depends on the dipole on the surface. The results in Figure 2 show the depletion of electrons from the ITO surface and the accumulation of electrons around Cl and F adatoms. Irradiating DAEs with UV light at different wavelengths provides two different isomers and changes the molecule structure from an open loop to a closed loop. Charge density exhibits a similar distribution for both the open and closed forms.

##### 3.1.1.3. Geometry and Bonding Mode

**Figure 3:**
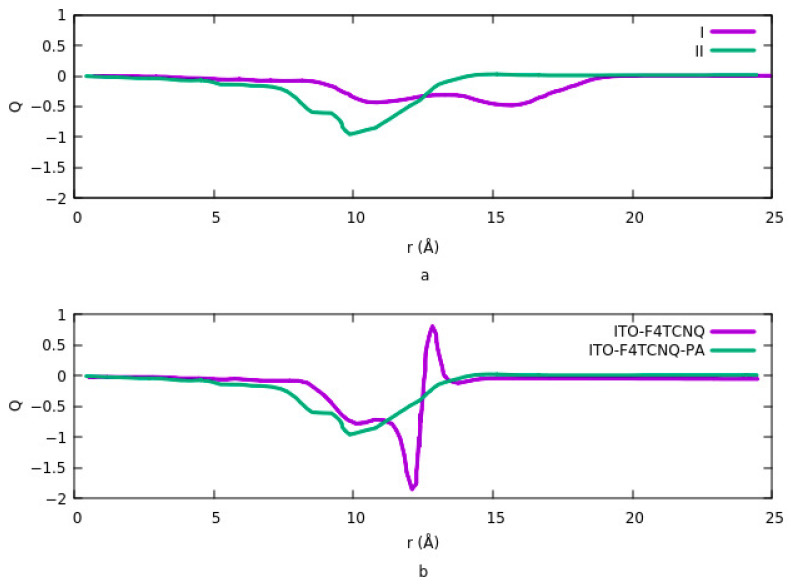
(**a**) Net change transfer for both configurations of adsorbing F_4_TCNQ to ITO. A large negative value in configuration II indicates that the charge transfer rate is higher when F_4_TCNQ is directly bound to ITO. (**b**) net charge transfer from ITO to F_4_TCNQ in the absence (purple) and presence of PA (green). The charge transfer rate is higher when F_4_TCNQ is directly bound to ITO.

To investigate the effects of organic molecules with different dipole sizes on work function, Timpel et al., in another study, investigated the hole or electron injection mechanism on the surface of the ITO [54]. Their model was based on an ITO slab, t-butyl-carbazole-substituted phosphonic acid molecules, which were chemisorbed as a monolayer on the surface, and F_4_TCNQ molecules were selected as a layer for physiosorbed binding to ITO. They designed two adsorption modes for binding F_4_TCNQ to PA-modified ITO: (1) F_4_TCNQ is adsorbed on top of the carbazole-PA molecule and indirectly binds to ITO and (2) direct physiosorbed binding of F_4_TCNQ to ITO. The reported binding energy between PA and F_4_TCNQ to the ITO is 1.6 eV and 0.98 eV, respectively. The charge transfer diagram between ITO and F_4_TCNQ for two configurations, as well as in the absence and presence of PA, is shown in Figure 3.

The changes in the charge transfer rate by moving from the surface of the ITO toward the biomolecules are illustrated in Figure 3. The distance (*r*) is defined in such a way that *r* = 0 Å is the bottom layer of the ITO slab which in total contains three layers of atoms, and for the topmost atom in the third ITO layer r is 9 Å. In configuration I, the width of the PA monolayer on the surface of the ITO is 5.5 Å. The distance between the topmost atoms in PA and F_4_TCNQ is 3.69 Å. In configuration II, the distance between the topmost atom in the F_4_TCNQ and the top oxygen atom in the ITO is 3.2 Å. Calculating the charge transfer at the interface for two configurations (Figure 3a) clearly illustrates that the charge transfer rate is higher in configuration II, where PA and F_4_TCNQ form a monolayer on the surface of the ITO. In this case, F_4_TCNQ is on the surface of the ITO with physiosorbed binding besides the PA, which is adsorbed on the surface with chemisorb binding. Figure 3b compares the binding of F_4_TCNQ to the surface of the ITO in the presence and absence of PA. It indicates that the physiosorbed-based binding of F_4_TCNQ to the surface of the ITO in the absence of the PA has almost the same effect on the charge transfer rate as to the case where F_4_TCNQ is on the surface in the presence of chemisorbed binding of the PA (configuration II). In addition, the results from comparing the charge transfer rate between the ITO surface and F_4_TCNQ and PA separately shows higher charge transfer rate occurs when F_4_TCNQ is adsorbed on the surface of the ITO.

The following can justify the difference in the charge transfer rate among the ITO surface and various organic molecules:

- Nature of binding modes between different molecules and the surface. Chemisorb- and physisorb-binding modes have different effects on the interface dipole moment. In this case, absorption of PA on the surface of the ITO takes place through the hydrogen binding between hydroxyle groups on the surface and phosphonate moiety ($PO_{3}^{2-}$) [110]. On the other hand, F_4_TCNQ is adsorbed through charge reorganization or charge transfer between the molecule and the surface. F_4_TCNQ is a p-type dopant with a strong electron-accepting ability. Its LUMO level is −5.2 eV which is very close to the HOMO level of many semiconductors. This makes doping feasible by charge transfer from the semiconductor materials to the dopant molecule [141].
- Change in electrostatic potential energy at the interface. Interface potential energy is defined as a bond dipole energy [142]. When F_4_TCNQ is directly bound to the ITO, the potential energy is much higher than the adsorption energy between PA and ITO [143]. Strong electrostatic interaction between F_4_TCNQ and the surface of the ITO induces a significant electron transfer between the F_4_TCNQ molecule and the surface of the ITO.
- Change in geometry and structure. The geometry of the F_4_TCNQ molecule and surface of the ITO is also dependent on the bond dipole. The cyano groups of the F_4_TCNQ are strongly twisted. Moreover, the electron distribution of the ITO could vary strongly with such changes in the geometry. This could result in a higher charge transfer rate for the case of physisorb-based binding of F_4_TCNQ to the surface of the ITO [144].

In another study, Wang et al. [89] modified the surface of ITO using PA with the incorporation of diarylethenes (PA-DAEs). DAEs can alter between two different isomers while being irradiated at different UV light wavelengths. Illuminating light (UV), changes the molecule structure from an open loop to a closed loop. This changes the electronic properties of the organic molecule. They used the same parameters of DFT calculation used in the previous works performed to study the modifications of the ITO surface with PA explained in the previous section. The DFT calculated work functions for open and closed forms are different. However, this difference reduces when the coverage density of the organic molecule is increased. This is due to the differences in depolarization effects for the two forms [145]. On the other hand, using closed or open form provide similar results for charge transfer and charge density difference (Figure 2c). Their results also suggest that charge transfer is mostly between the ITO and PA. No significant charge transfer takes place between the ITO and DAE or PA and DAE.

##### 3.1.1.4. Bond Length and Charge Transfer Rate

The effects of halogenation of ITO on its work function was studied by Hung et al. [91]. Their focus was to understand the origin of the high work function of chlorinated ITO. They defined three adsorption sites: top site, above a metal atom, bridge site is between two adjacent metal atoms and a hollow site which is at the center of the quadrilateral grid. The work function is dependent on surface coverage, the geometry of the bonding and adsorption sites. At low coverage density, the most stable geometry is for the top site and increasing the coverage density shifts the bridge site to the most stable site. It also increases the potential energy step since the number of diploes increases as such work function is linearly increased in this case. They suggested that electronegativity and the atomic size of the halogen are two factors that must be considered simultaneously to explain the change in the dipole. The charge density difference for the Cl-ITO system indicates a depletion of electrons from the ITO surface and the accumulation of electrons around the Cl atom. This is due to a surface diploe layer induced by charge transfer in the system. They repeated the calculations using fluorine (F) instead of Cl. Although F is the most electronegative and reactive element, however, the change in charge density difference and work function when doping ITO with F is lower compared to the Cl-ITO system (Figure 2d). The reason is the lower diploe moment in the F-ITO system. Dipole moment is related to the number of charges and the distance between the charges. The charge distribution depends on the difference in electronegativity of the atoms. The distance is related to the size of the adatom and the surface metal atom. Since F has a smaller atomic radius than F, therefore the charge-transfer distance is smaller for the F-ITO system. This effect overcomes the Fermi level or electrochemical potential of the ITO. Consequently, the charge transfer distance is the main parameter for evaluating the change in work function.

### 3.2. Effects of Surface Modification on Potential Energy

**Figure 4:**
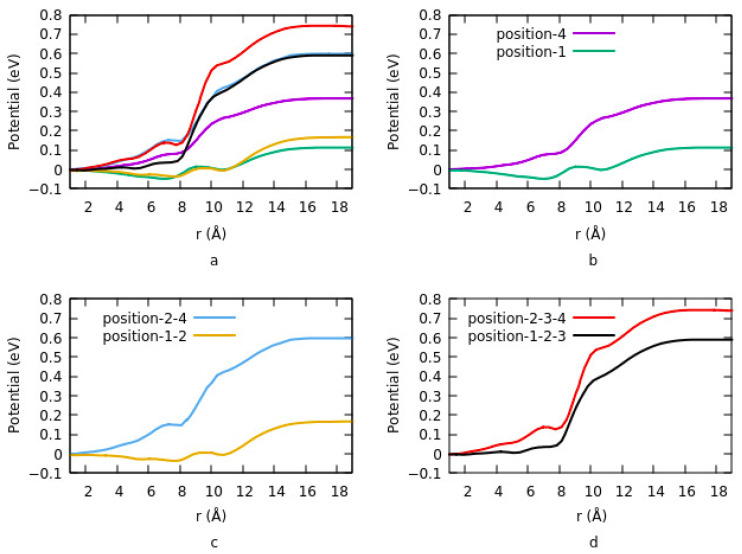
(**a**) Change in potential energy for different adsorption geometry and coverage density for adsorption of phosphonic acid. (**b**) one molecule per unite cell and at site 1 and site 4. (**c**) for two molecules per unite cell and at sites 1, 2, and 2, 4. (**d**) for 3 molecules per unit cell and at two different configurations of 1, 2, 3 and 2, 3, 4.

The vacuum potential energy change is one of the components determining work function when ITO is modified by an organic molecule. Effects of coverage density, size, bond length, and charge transfer on potential energy investigated in different studies [66,110]. Vacuum potential is directly proportional to the dipole moment. Based on the work by Li et al., the potential energy has similar behavior with the charge difference density at various coverage densities and geometry. Figure 4 shows the change in potential at different sites and having different geometries. Details are explained in Section 3.1.1.2. For lower coverage densities, vacuum potential or bond dipole depends on the site of adsorption. In summary, the existence of two opposite dipole moments across the interface results in canceling out each other at a geometry. The lowest potential is obtained in this case. On the other hand, the highest potential corresponds to an equivalent single dipole moment.

**Figure 5:**
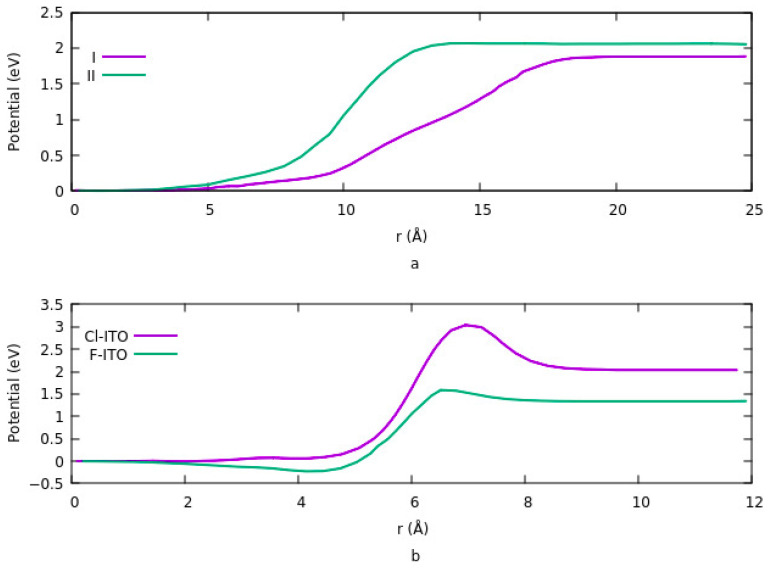
Comparing the vacuum potential for two different systems. (**a**) functionalizing the ITO with PA-F_4_TCNQ in two different modes when F_4_TCNQ is bin to the ITO in the presence of the PA (I) or directly bind to the ITO (II). (**b**) Effects of halogenation on vacuum potential.

Effects of the size on interface dipole similarly provide the same results that were explained for charge transfer rate and charge density differences. Figure 5a shows the potential energy for two different configurations when the ITO is modified with PA-F_4_TCNQ. The vacuum potential step occurs due to the dipole moment at the interface. In configuration 1, the step is the sum of two different dipole moments, and in configuration 2, it is the net dipole moment of the combination of PA and F_4_TCNQ. Since the charge transfer rate is higher in the second mode, therefor the potential energy is also higher in this case. In addition, the system goes to saturation sooner in mode 2. In Figure 5b, the effect of halogenation on potential is represented. The vacuum potential has the same pattern as the work function. As it is clear, modifying ITO with Cl induces a larger step in the system compared to F. The reason is explained in Section 3.1.1.4. dipole moment, which is related to the magnitude of the charge transfer rate and the distance between the charges in ITO and electronegative element is higher in the case of Cl compared to F.

### 3.3. Effects of Surface Modification on the Density of State

**Figure 6:**
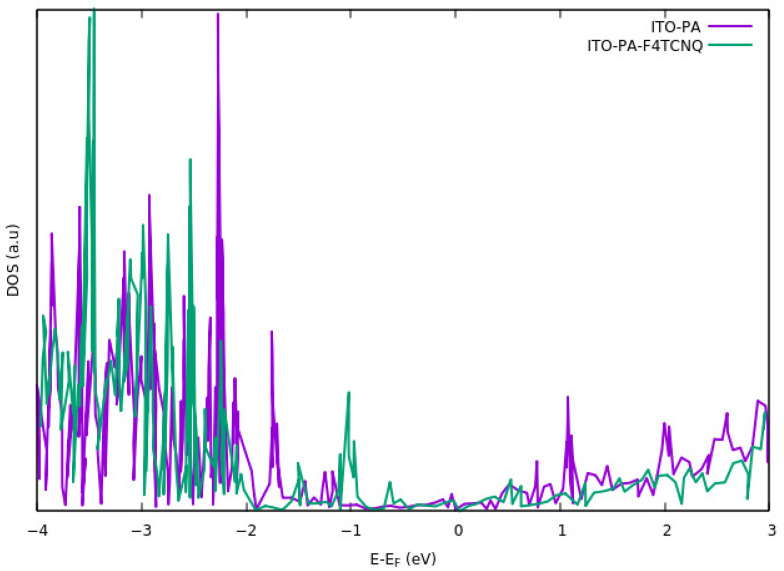
DOS for ITO modified with only PA and PA in the presence of F_4_TCNQ. For both cases, the shift in the main peaks in the valence band is visible. The shift is larger when the F_4_TCNQ is also bound to the ITO.

To study the electrons and holes in the ITO with organic molecules on the surface, the density of states (DOS) of molecules were analyzed [66,89]. Hong et al. [66] studied the energy alignment of HOMO and LUMO levels of the PA-F_4_TCNQ on the surface of ITO. They stated that adsorbing F_4_TCNQ results in shifting the HOMO level in the carbazole-PA molecule up to the ITO Fermi level. Figure 6 shows the DOS for the PA-ITO system and PA-F_4_TCNQ modified on ITO. in both cases, there exists a shift in the valence band. The shift is larger when the F_4_TCNQ is directly bound to the ITO. Their results also suggest that the LUMO energy of the F_4_TCNQ is aligned with the ITO Fermi level. Shift in HOMO level of carbazole-PA, plays the major role regarding charge injection mechanism at the interface of ITO-PA-F_4_TCNQ. On the other hand, alignment of LUMO level in F_4_TCNQ results in the formation of a hole transport layer.

Wang et al. [68] analyzed the valence electron region of the ITO modified by PA-DAE. Based on the UPS characterization, a change in DOS of the system is observed by using DAE open and closed form. Their results indicate a shift in HOMO level when the DAE is illuminated using UV light.

### 3.4. Effects of Surface Modification on Catalytic Properties

In this section, we explain the catalytic properties of nanostructured ITO after surface modification. Nanostructured materials have applications in different fields such as energy storage, sensing, and biosensing, green chemistry, drug delivery, etc. [146,147,148,149]. Nanostructured ITO, due to its electrocatalytic properties, is a promising material in chemical sensors and the early-stage detection point of care biosensors [17,137]. Most of the experimental works are based on thin-film ITO. The reason is the application of thin-film in catalytic properties. Nanostructured ITO thin film can be produced by coating a layer of ITO in the range of less than a micron. Properties of the deposited film, such as topography, morphology, grain size, etc., affect the catalytic behavior of the ITO [150]. Increasing sensitivity, providing high surface area, and therefore improving the limit of detection are some of the aspects of using nanostructured ITO with a thickness of less than 200 nm. According to experimental work, atomic force microscopy (AFM) images taken from ITO thin film indicate that grain and pore size of the ITO film increase by increasing the thickness of the deposited film [151]. X-ray diffraction patterns for samples with different thicknesses indicate that by increasing the thickness, the lattice constant decreases while grain size increases. Better crystallinity is also achieved in the case of higher film thickness [51]. The conductivity of the ITO thin film has a direct relation with the thickness. Studies show that increasing thickness up to 100 nm increases the conductivity of the ITO thin film [152,153].

#### 3.4.1. Effect of Thickness on Properties

**Figure 7:**
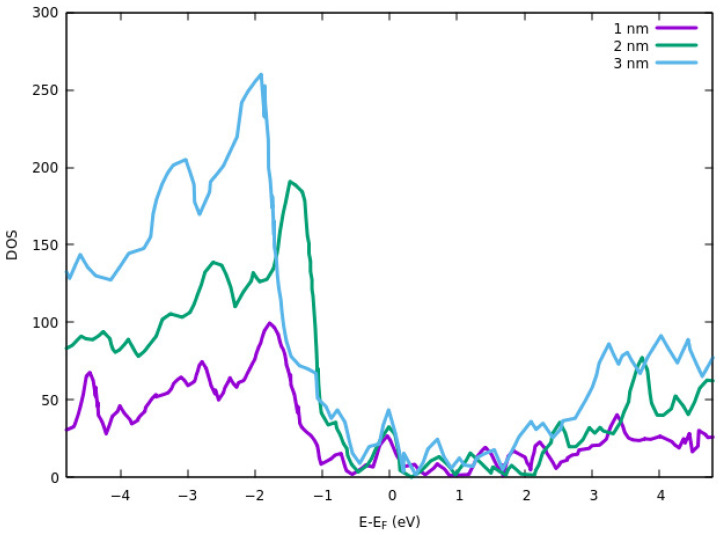
DOS of ITO thin films for different thicknesses.

The electrical resistivity of the ITO films at different thicknesses was calculated using DFT simulations by Liu et al. [87]. The calculations were performed by employing VASP. 400 eV cut-off plane wave basis set and energy threshold of 10^−4^ eV. GGA exchange-correlation functional of PBE and PAW method was used to investigate the electronic properties and core-electron interactions. DOS results for different thicknesses are shown in Figure 7. The DOS peak for the valence band is higher for all three peaks than the conduction band. The reason for the smaller DOS of 1 nm thickness ITO in the conduction band is due to partial hybridization of the antibonding orbitals. The electrical resistivity of the films was also calculated computationally. They showed that the thinner layer has a higher resistivity. Higher resistivity at a lower thickness of the ITO is attributed to the strain effect. The average strain is increased with decreasing the thickness. This results in a shorter bond length and a lower free-electron path that ultimately results in higher resistivity.

The transmittance of ITO at the temperature of *T* = 300 K was also investigated using the DFT method [87]. The transmittance was calculated based on the relation between reflectance (*R*), adsorption coefficient (*α*) and thickness of the film (*t*). According to Equation (1) [87], the transmittance can be defined as:

(1) $$T=\left(1-R\right)e^{-\alpha t}$$

**Figure 8:**
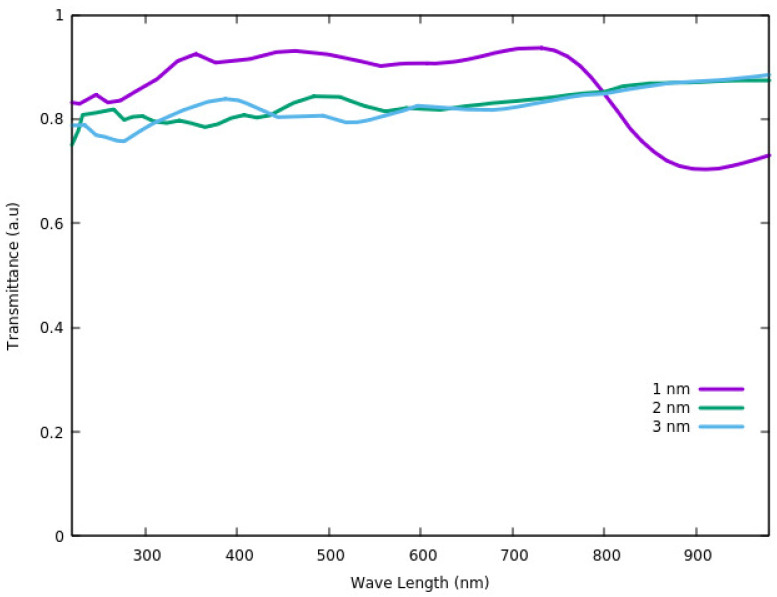
Transmittance spectra of ITO thin films.

Based on Figure 8, the largest transmittance in the visible region (<780 nm) is for the thickness of 1 nm. Considering the wavelengths of >780 nm, which is in the range of near infra-red region (NIR), the lowest thickness has the smallest transmittance. As such we can conclude that for ultra-thin ITO films (most probably for thin films under 10 nm), the transmittance depends on the size but shows two different behaviors in visible and NIR regions. It increases with increasing the thickness in the NIR region and shows reverse behavior in the visible light region [87].

According to Liu et al. [87], the bandgap of 1 nm ITO is about 0.8 eV, which results in the semiconducting nature of the ITO thin film. The ITO with a thickness of 2 nm has a band gap of 0.1 eV. For ITO with 3 nm thickness, they indicate that the band gap is very narrow due to extra bond occupations near the Fermi energy.

#### 3.4.2. Oxygen and Hydroxyl Functional Groups

In nanostructured metal oxide materials, surface properties such as reactivity are significantly important compared to properties of bulk material. Based on the synthesis process, the surfaces can be metal or oxygen terminated and, in some cases, a combination of both. In a study performed by Zhou et al. [21], it is shown that oxidation of the ITO surface takes place when the surface is metal-terminated. In other words, metal terminated ITO is unstable when exposed to O_2._ As such, the oxygen atom covers the whole surface and provides a metal oxide terminated surface. These surfaces are unstable while exposed to oxygen and/or water molecules. Small activation barriers are required for the chemisorption binding between the surface atoms and O_2_ or H_2_O molecules. Based on the results, O_2_ chemisorption is the dominant reaction at low surface coverage and generates surface dimers. Increasing the number of dimers on the surface changes the metal terminated to the oxygen-terminated surfaces. Thus, the chemisorption of oxygen atoms on the surface substitutes with the dissociative chemisorption process of water. Hydroxyls form on the surface of the ITO due to the dissociative chemisorption of H_2_O.

#### 3.4.3. Carboxylate Functional Groups

In a study performed by Chen et al. [88], it was shown that modifying the ITO with trimesic acid (TMA) leads to the formation of carboxylate groups on the TMA-modified ITO nanoparticles. They performed computational results to find the preferred adsorption configuration of TMA on the ITO. Vertical adsorption is reported to be the most kinetically favorable. Direct modification of ITO is thermodynamically favorable in this case as well. Based on the DFT calculations, the TMA molecule binds to the ITO (111) surface with an upstanding configuration employing dissociative chemisorption effect. This effect leads to the formation of a covalent bond between oxygen in the carboxylate group of TMA and indium on the ITO surface. Hydrogen bonding also forms due to the interaction of hydron in the carboxylate groups with the nearest oxygen atom. The formation of carboxylate functional groups provides a catalytic-active molecule. The ability of high charge transfer makes the TMA-ITO able to participate in redox reaction and optoelectronic applications.

Rittich et al. [23] investigated the effects of carboxylic acid self-assembled monolayers on the work function of ITO. Based on their results, functionalization of the ITO with amino acids decreases the work function significantly. Carboxylic acid decreases the barrier energy, while the transparency and other properties are not affected. The reduction is due to variation in the type of spacer and head groups of the carboxylic acid. They also studied the effects of spacer chain length on the wok function and concluded that there is no change in work function by increasing the chain length of the molecule. This demonstrates that coupling between anchor and head group provided by the spacer has a direct relation with delocalized electron and consequently on work function.

## 4. Conclusions and Prospects

Indium tin oxide is an interesting metal oxide for researchers due to its unique optical and electrical properties. Its surface and structural properties in different forms, such as thin-film and nanocomposite, have been investigated in both experimental and theoretical works. Based on different studies, it is shown that the best method to characterize the properties of the ITO theoretically is using DFT and that using VASP is faster than other packages for these studies. Choosing the parameters such as geometrical structures and energies based on the experimental data results in a good agreement between theoretical and experimental results.

Modification of the ITO surface with organic molecules is an interesting approach toward enhancing its surface properties through charge transport between the surface of the ITO and the organic layer. Among different molecules functionalized on the surface of the ITO so far, it is reported that F_4_TCNQ has the strongest effects on the surface properties of the ITO. It is shown that physisorption binding of the F_4_TCNQ in the presence and absence of PA affects the charge density extremely and, ultimately, the work function of the ITO. Studies are explaining the role of the dipole moment of organic molecules as the main factor on properties of the ITO in detail. It is shown that coverage density and binding geometry are two main parameters affecting the dipole. However, the studies conducted so far do not provide any insights toward the effects of changing the surface properties on the underneath layers in the ITO structure. As such, a potential field of study is to investigate the properties of ITO in bulk structure due to surface modification. Covering this gap will let us know how the effects on the surface gradually affect the properties and transformations under the surface.

On the other hand, reports indicate that the electrical properties of the ITO surface change by modifying the surface of the ITO with inorganic molecules such as halogens as well. In this case, the two main parameters affecting properties such as charge density and work function are the magnitude or number of charges and the distance between the charges. The first parameter depends on the difference in electronegativity of the atoms. The second is due to the size of the adatom and the surface metal atom. The larger these two parameters are, the larger the dipole moment would be, which results in a higher work function. Studies have also been performed on the effects of parameters such as chain lengths and aromaticity of hydrocarbons on structural properties of functionalized ITO. It is reported that intermolecular interaction is not significantly affecting the dipole moments.

Catalytic properties of the ITO, along with its other physical properties, significantly change with the surface modifications. Based on our review, the formation of various functional groups such as oxygen, hydrogen, and carboxylate functional groups on the surface of nanosized ITO results in increasing catalytic activities of the ITO. In this case, surface modification influences the ITO surface properties such as reactivity. In the case of optoelectronic properties of ultrathin film ITO, computational results of band gap and DOS of the ITO after surface modification are in good agreement with the experimental data. Increasing the hydroxylation on the surface of the nanostructured ITO induces the shift in DOS toward the valence band. Moreover, ultrathin films with 1 nm thickness have a larger band gap and transmittance in the visible light region compared with that of 2 and 3 nm ITO films. However, there is still a gap in theoretical studies of optoelectronic properties of the nanostructured ITO as a function of film thickness. The effect of size on the properties of the nanostructured ITO could be an attractive topic for researchers to study.

## Figures and Tables

**Table 1:** Different parameters affecting work function of ITO in the presence of functional groups.

	Modifiers	Experimental/Computational Method	Description	References
1	Providing M-terminated surface	Computational	Vacancy decreases the work function	[21]
2	Providing O-terminated surface	Computational	Vacancy does not change the work function	[21]
3	OH	Computational	The OH coverage increases the work function	[21]
4	Crystallographic structure	Computational	In BCC structure, vacancy decreases the work function	[21]
5	Carboxylic acid	Experimental and computational	By increasing dipole moment, the work function increases	[23]
6	Carboxylic acid	Experimental	Chain length does not affect the work function	[23]
7	Carboxylic acid	Experimental	Work funtion changes by varying the amount and orientation of alliphatic groups	[23]
8	Phosphonic acid	Experimental and computational	By decreasing the dipole moment, the work function increases	[46]
9	PA-F_4_TCNQ	computational	Doping increases the work function of PA-F_4_TCNQ -ITO	[54]
10	F_4_TCNQ	Computational	Doping increases the work function of F_4_TCNQ-ITO	[54]
11	PA-DAE	Experimental and computational	Doping increases the work function of PA-DAE-ITO	[89]
12	Cl doping	Computational	By increasing Cl coverage, the work function increases	[91]
13	F doping	Computational	By increasing F coverage, the work function increases	[91]
14	UV exposure	Experimental	Work function decreases by exposure time	[131]
15	Air exposure	Experimental	Work function increases by exposure time	[131]
16	O_2_ exposure	Experimental	Work function increases by exposure time	[131]

## Data Availability

Relevant data are available from the authors on request.
